# The Healing of Arteries after Ligature in Man and Animals

**Published:** 1886-12

**Authors:** 


THE HEALING OF ARTERIES AFTER LIGATURE IN MAN AND ANIMALS. *By* J. COLLINS WARREN, M.D., *Assistant Professor of Surgery, Harvard University; Surgeon to the Massachusetts General Hospital; Member American Surgical Association; Honorary Fellow Philadelphia Academy of Surgery. One volume, pp. 184, illustrated with twelve full-page plates in black and colors. Parchment-muslin binding. New York:* WILLIAM WOOD & COMPANY.

The author claims that this book represents what he has observed in an attempt "to observe not only the behavior of the various tissues concerned in the process of repair, but also the different phases through which the vessel passes from the moment of ligature until the condition is reached after which no further change occurs."

The book is divided into five chapters, viz., History, Experiments on Animals, Human Subject, Closure of the Fœtal Vessels, and Summary. All that the author has to say will be read with interest by pathologists and surgeons, but that which is of greatest interest to the general professional reader is contained in the summary, which is so concisely written as not to permit further condensation. However, mere mention of a few of the conclusions reached will be of interest in this notice of the book. The period of time after ligature to the development of the final scar, varies with different vessels, but in no case was it observed to be less than three months. Another prominent feature observed is the complex nature of the process. Many museum specimens examined had the appearance of union by adhesion, but careful examination did not justify such conclusion. The experiments "seemed to show that the muscular cell is a prominent and essential feature of the arterial cicatrix. The size of the thrombus depends upon *a*, the amount of traumatism, and *b*, the presence of aseptic conditions, it being always present, Senn and Baumgarten to the contrary nevertheless, and large in direct proportion to the amount of traumatism to the artery itself and to the amount of suppuration in the wound. It is probable that an hour or more may elapse before the thrombus has become perceptible to the naked eye.

True progress in the advancement of medical knowledge is made by just such work as this book represents, and the manner and matter of the report is beyond criticism. The paper, printing and binding, and the careful accuracy of the drawing of the illustrations, combine to make the book a very handsome and exceptionally fine volume.

